# Distinct domains in Bub1 localize RZZ and BubR1 to kinetochores to regulate the checkpoint

**DOI:** 10.1038/ncomms8162

**Published:** 2015-06-02

**Authors:** Gang Zhang, Tiziana Lischetti, Daniel G. Hayward, Jakob Nilsson

**Affiliations:** 1 The Novo Nordisk Foundation Center for Protein Research, Faculty of Health and Medical Sciences, University of Copenhagen, Blegdamsvej 3B, 2200 Copenhagen, Denmark

## Abstract

The spindle assembly checkpoint (SAC) ensures proper chromosome segregation by delaying anaphase onset in response to unattached kinetochores. Checkpoint signalling requires the kinetochore localization of the Mad1–Mad2 complex that in more complex eukaryotes depends on the Rod–Zwilch–ZW10 (RZZ) complex. The kinetochore protein Zwint has been proposed to be the kinetochore receptor for RZZ, but here we show that Bub1 and not Zwint is required for RZZ recruitment. We find that the middle region of Bub1 encompassing a domain essential for SAC signalling contributes to RZZ localization. In addition, we show that a distinct region in Bub1 mediates kinetochore localization of BubR1 through direct binding, but surprisingly removal of this region increases checkpoint strength. Our work thus uncovers how Bub1 coordinates checkpoint signalling by distinct domains for RZZ and BubR1 recruitment and suggests that Bub1 localizes antagonistic checkpoint activities.

## Abstract (web summary)

The spindle assembly checkpoint (SAC) depends on the recruitment of specific protein complexes to the kinetochore. Here Zhang *et al*. show that Bub1 recruits the RZZ complex and BubR1 to the kinetochore, and loss of the BubR1 binding sequence enhances checkpoint activity suggesting both SAC activating and silencing roles.

## Graphical abstract

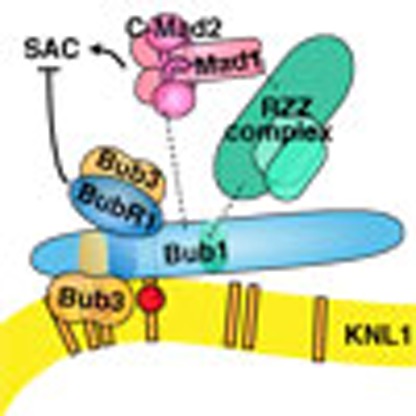

Proper segregation of sister chromatids during mitosis is ensured by the spindle assembly checkpoint (SAC). In response to incorrectly attached kinetochores, the SAC delays progression into anaphase by inhibiting Cdc20, the mitotic co-activator of the Anaphase Promoting Complex/Cyclosome (APC/C)^1^,^2^. The SAC is composed of a set of conserved checkpoint proteins Bub1, BubR1 (Mad3 in yeast), Bub3, Mad1, Mad2 and the Mps1 and Aurora B kinases^2^. Bub1 and BubR1 are both bound to Bub3 while Mad2 exists both in a soluble form and in a stable complex with Mad1 (Mad1/2). Cdc20 is inhibited by the direct binding of Mad2 and BubR1 to form the mitotic checkpoint complex (MCC).

The kinetochore acts as a signalling scaffold for the SAC. Indeed, all the checkpoint proteins associate dynamically with the kinetochore, with Bub1 and Mad1 being more stably bound than the other checkpoint proteins^3^,^4^. With the increased understanding of the kinetochore, it has become clear that the KNL1-Mis12-Ndc80 (KMN) network is the binding site not only for microtubules but also for checkpoint proteins^5^,^6^,^7^. In particular, the large KNL1 protein appears to be a platform for recruitment of SAC proteins. One of the earliest events upon activation of the checkpoint is that Mps1 phosphorylates numerous so-called MELT repeats. The phosphorylated MELT repeats then act as direct binding sites for the Bub1–Bub3 complex^8^,^9^,^10^,^11^,^12^,^13^,^14^,^15^. The recruitment of Bub1 to kinetochores, in turn, facilitates the recruitment of BubR1 by an unknown mechanism^16^,^17^. BubR1 kinetochore localization also depends on its association with Bub3 and mutation of the Bub3 binding site in BubR1 impairs both its kinetochore localization and checkpoint signalling^18^,^19^,^20^. The fact that BubR1 needs to localize to kinetochores for a functional checkpoint suggests that its assembly into the MCC occurs at kinetochores. However, a number of labs have also provided evidence that BubR1 kinetochore localization is not critical for a functional checkpoint^21^,^22^,^23^. In addition, recent data have implicated a novel role of BubR1 in SAC silencing through its direct binding to the B56–PP2A phosphatase to initiate dephosphorylation of the MELT repeats either directly or through stimulating protein phosphatase 1 binding to KNL1 (refs 24, 25, 26, 27, 28, 29). In addition, Bub3 has been shown to be important for SAC silencing but not SAC signalling in fission yeast suggesting that kinetochore localized Mad3–Bub3 contributes to SAC silencing^30^.

In addition to recruiting BubR1 to kinetochores, Bub1 also stimulates Mad1/2 kinetochore localization^16^,^17^,^31^. In yeast and worms, the Mad1/2 complex is recruited to kinetochores by a direct interaction with Bub1 but such a direct interaction has not been identified in human cells^31^,^32^,^33^. The lack of a stable Bub1–Mad1/2 interaction in human cells could be due to the presence of the Rod–Zwilch–ZW10 (RZZ) complex that is only present in complex eukaryotes and contributes to Mad1/2 kinetochore localization and checkpoint signalling^34^,^35^,^36^,^37^. No direct interaction between the RZZ complex and the Mad1/2 complex has been reported and the role of the RZZ complex in Mad1/2 kinetochore localization is unclear. The RZZ complex is bound to the dynein complex through the adaptor protein Spindly and the dynein complex can strip the RZZ from kinetochores through minus end-directed transport along kinetochore microtubules^38^,^39^,^40^,^41^,^42^,^43^. This dynein-mediated stripping also removes the Mad1/2 complex and contributes to checkpoint silencing but Mad1/2 can also be removed by a dynein-independent mechanism^38^,^40^.

The RZZ complex has been proposed to be recruited to kinetochores via a direct interaction between the ZW10 subunit of the complex and the outer kinetochore protein Zwint^44^,^45^. However, the identification of ZW10 mutants that fail to bind Zwint yet still localize to kinetochores, although less stably, suggests that additional kinetochore components contribute to RZZ localization^46^.

Given the importance of localizing RZZ and BubR1 to kinetochores for checkpoint signalling, we here set out to examine the molecular mechanisms of their recruitment. Surprisingly, we find that RZZ kinetochore recruitment does not depend on Zwint but instead depends on Bub1. We define a region in the central part of Bub1 necessary for RZZ localization and for checkpoint signalling. Furthermore, we identify a distinct domain in Bub1 that can bind directly to BubR1 and is necessary for Bub1-dependent kinetochore recruitment of BubR1. However, we find that this domain in Bub1 is not required for SAC signalling but could contribute to SAC silencing. Together, the data in this work provide novel insight into how checkpoint proteins are recruited by Bub1 to kinetochores and suggests that Bub1 recruits both SAC signalling and silencing activities to make the checkpoint dynamic.

## Results

### Kinetochore recruitment of RZZ independent of Zwint

**Figure 1:**
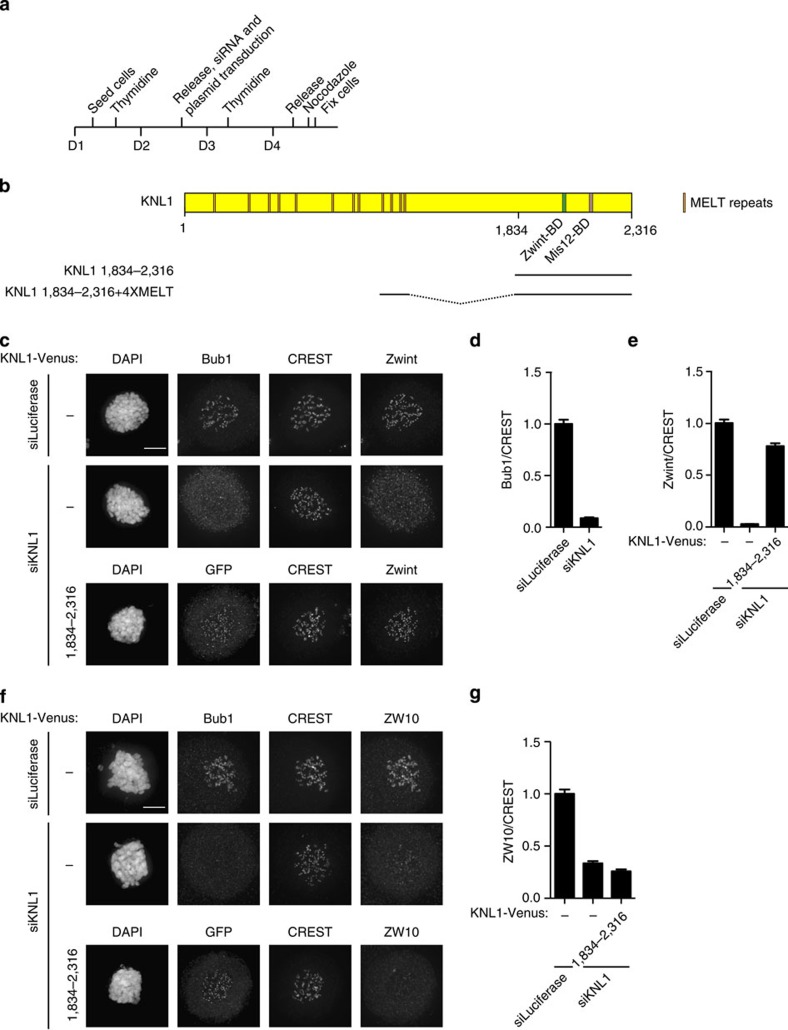
ZW10 kinetochore localization is not supported by a KNL1 fragment containing the Zwint binding domain. (**a**) Outline of the synchronization protocol used in this study. Cells were fixed 2 h after addition of nocodazole. (**b**) Schematic of human KNL1 with functional regions indicated. The region encoded by KNL1 1,834–2,316 and KNL1 1,834–2,316+4XMELT is depicted below. (**c**) HeLa cells were treated with a control RNAi oligo (luciferase) or an RNAi oligo targeting KNL1 for 48 h and arrested in mitosis using nocodazole for 2 h. In one condition, cells were co-transfected with a plasmid encoding KNL1 1,834–2,316-Venus as indicated. Cells were fixed and stained with DAPI, CREST (centromere marker) and Zwint. We used Bub1 as a KNL1 depletion marker because both our KNL1 and Zwint antibodies are rabbit polyclonal. Bub1 solely depends on KNL1 for its localization and its kinetochore levels follow KNL1. The expression of KNL1 1,834–2,316 Venus was detected by staining cells with a GFP antibody. (**d**,**e**) The kinetochore levels of Bub1 and Zwint were determined in the indicated conditions and normalized to CREST. (**f**,**g**) As in **c**–**e**, but cells were stained with a ZW10 antibody and the kinetochore levels of ZW10 were measured and normalized to CREST in the indicated conditions. At least 160 single kinetochores from eight different cells were measured in all the conditions and the mean with standard error of mean is indicated. Scale bar, 5 μm.

As described in the introduction, the role of Zwint in RZZ localization is not consistent, therefore, we decided to examine the role of Zwint in more detail. We first established an RNAi depletion protocol for Zwint. HeLa cells were synchronized using a double thymidine block protocol and cells were treated with RNA interference (RNAi) oligos immediately after the release from the first block (Fig. 1a). Six hours after release from the second thymidine, block cells were treated with nocodazole for 2 h and then fixed and analysed by immunofluorescence using a deconvolution microscope. This synchronization protocol ensures that we only observe the first mitosis after Zwint depletion. The inclusion of nocodazole prevents indirect effects on kinetochore localization due to differences in kinetochore–microtubule interactions. Zwint kinetochore levels were measured from the three consecutive z-stacks, each 200 nm apart, encompassing the bulk of kinetochore signals and normalized to a constant centromere marker (CREST). Visual inspection revealed efficient transfection with >90% of cells displaying a robust depletion of Zwint. Using this approach, Zwint was reduced to ∼20% of its normal levels at kinetochores in most mitotic cells (Supplementary Fig. 1a,b).

As recently observed by the Salmon Lab, we found that depletion of Zwint by RNAi reduced the kinetochore levels of the KNL1 kinetochore protein^7^ (Supplementary Fig. 1c,d). We also observed that the total KNL1 protein levels were reduced in mitotic cell extracts when Zwint was depleted, suggesting that KNL1 might become unstable if not bound to Zwint (Supplementary Fig. 1e). As KNL1 kinetochore levels could be restored by expressing an short interfering RNA-resistant version of Zwint, this argued against an RNAi off-target effect on KNL1 (Supplementary Fig. 1c,d).

**Figure 2:**
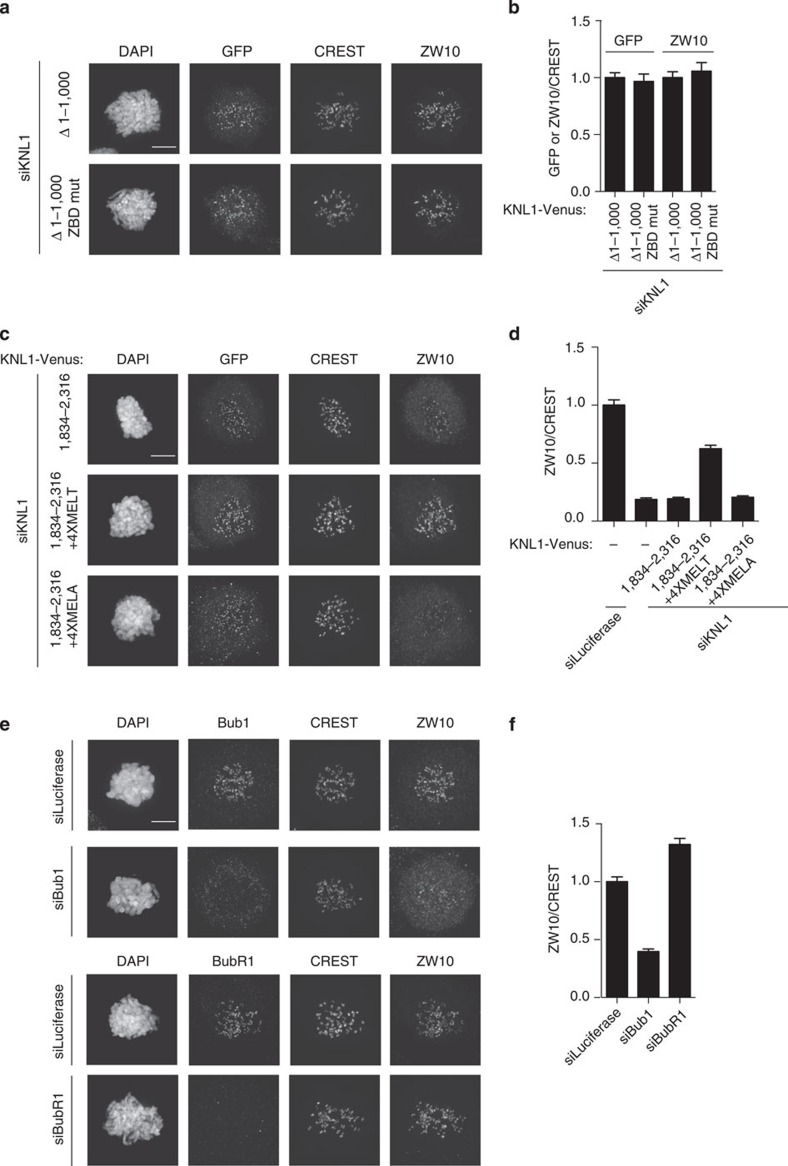
Bub1 is required for RZZ localization to kinetochores. (**a**) HeLa cells were depleted of endogenous KNL1 using RNAi and the indicated KNL1 constructs were co-transfected for 48 h and cells were arrested with nocodazole for 2 h. Cells were fixed and stained for GFP (KNL1 constructs), CREST and ZW10. (**b**) The ratios of ZW10/CREST and GFP/CREST signal were calculated. (**c**) As in **a** with the ZW10/CREST ratio shown in **d**. The GFP/CREST values for the three KNL1 constructs were: KNL1 1,834–2,316: 1.00; KNL1 1,834–2,316+4XMELT: 1.11; KNL1 1,834–2,316 4XMELA: 0.85. (**e**) HeLa cells were treated with luciferase, BubR1 or Bub1 RNAi for 48 h and cells were arrested with nocodazole for 2 h. Cells were fixed and stained for Bub1, BubR1, CREST and ZW10 as indicated. (**f**) The kinetochore levels of ZW10 were measured and normalized to CREST and at least 160 single kinetochores from eight different cells was measured in all the conditions and the mean with standard error of mean is indicated. Scale bar, 5 μm.

Given these observations, it was clear that the effects of Zwint RNAi might be indirect because of its effect on KNL1. We therefore decided to carefully reinvestigate whether Zwint was the only kinetochore receptor of the RZZ complex, as a number of studies had previously suggested. First, we depleted KNL1 by RNAi using the outlined protocol. This resulted in >90% depletion of KNL1 from kinetochores in most mitotic cells^12^. As expected, this fully prevented Bub1 and Zwint kinetochore localization (Fig. 1c–e). Next, we expressed Venus-tagged RNAi-resistant KNL1 residues 1,834–2,316 by co-transfecting it with the KNL1 RNAi oligo (Fig. 1a). KNL1 1,834–2,316 contains both the Zwint binding domain and the kinetochore-targeting domain of KNL1 and indeed expression of this rescued Zwint kinetochore localization (Fig. 1b–e). Next, we examined RZZ kinetochore localization by using ZW10 kinetochore levels as readout for this. ZW10 kinetochore levels were reduced to ∼30% upon KNL1 depletion but this could not be restored by expressing KNL1 1,834–2,316 suggesting that Zwint itself cannot directly recruit the RZZ complex (Fig. 1f,g). To determine whether Zwint was required for RZZ recruitment, we mutated four amino acids to alanines in KNL1 (referred to as ZBD mut)(Supplementary Fig. 2a). These four amino acids have previously been shown to be required for KNL1 interaction with Zwint in a yeast two-hybrid assay^47^. For these experiments, we used a KNL1 construct that lacks the first 1,000 amino acids (KNL1 Δ1,000) but contains four MELT repeats that is sufficient for KNL1 functionality^12^,^13^,^14^. KNL1 Δ1,000 restored Zwint kinetochore localization after KNL1 RNAi but KNL1 Δ1,000 ZBD mut did not (Supplementary Fig. 2b,c). However, there was no difference in the ability of the two KNL1 proteins in recruiting ZW10 to kinetochores (Fig. 2a,b). In agreement with our observation that endogenous KNL1 became unstable when we depleted Zwint, the mutation of the Zwint binding domain in KNL1 Δ1,000 resulted in very low expression levels of this protein. However, by extensively screening many mitotic cells, we were able to identify cells expressing similar levels of KNL1 Δ1,000 ZBD mut and KNL1 Δ1,000 (Fig. 2b).

These results show that Zwint in itself cannot localize ZW10 to kinetochores and that removing Zwint from KNL1 does not affect ZW10 localization. This is inconsistent with a model whereby a direct Zwint–ZW10 interaction alone mediates RZZ kinetochore localization but consistent with the identification of ZW10 mutants that cannot bind Zwint yet still localize to kinetochores^46^. Our experiments do not address whether a Zwint–ZW10 interaction could help stabilize the RZZ complex on kinetochores but argue for additional KNL1-dependent activities in efficiently localizing RZZ. We set out to identify these next.

### Bub1 is required for RZZ kinetochore localization

Since KNL1 Δ1,000 could recruit ZW10 while KNL1 1,834–2,316 could not, we speculated that MELT repeats of KNL1 are required for RZZ recruitment. To test this, we fused amino acids 1,000–1,200 of KNL1, encompassing four MELT repeats, to KNL1 1,834–2,316 (KNL1 1,834–2,316+4XMELT). The addition of MELT repeats, but not the mutated MELT repeats (MELA), to KNL1 1,834–2,316 clearly stimulated its ability to recruit ZW10 to kinetochores, pointing to a role of the MELT repeats in kinetochore localization of the RZZ (Fig. 2c,d). Since the MELT repeats of KNL1 recruit both Bub1 and BubR1, we next investigated whether either of these checkpoint proteins was required for ZW10 localization.

**Figure 3:**
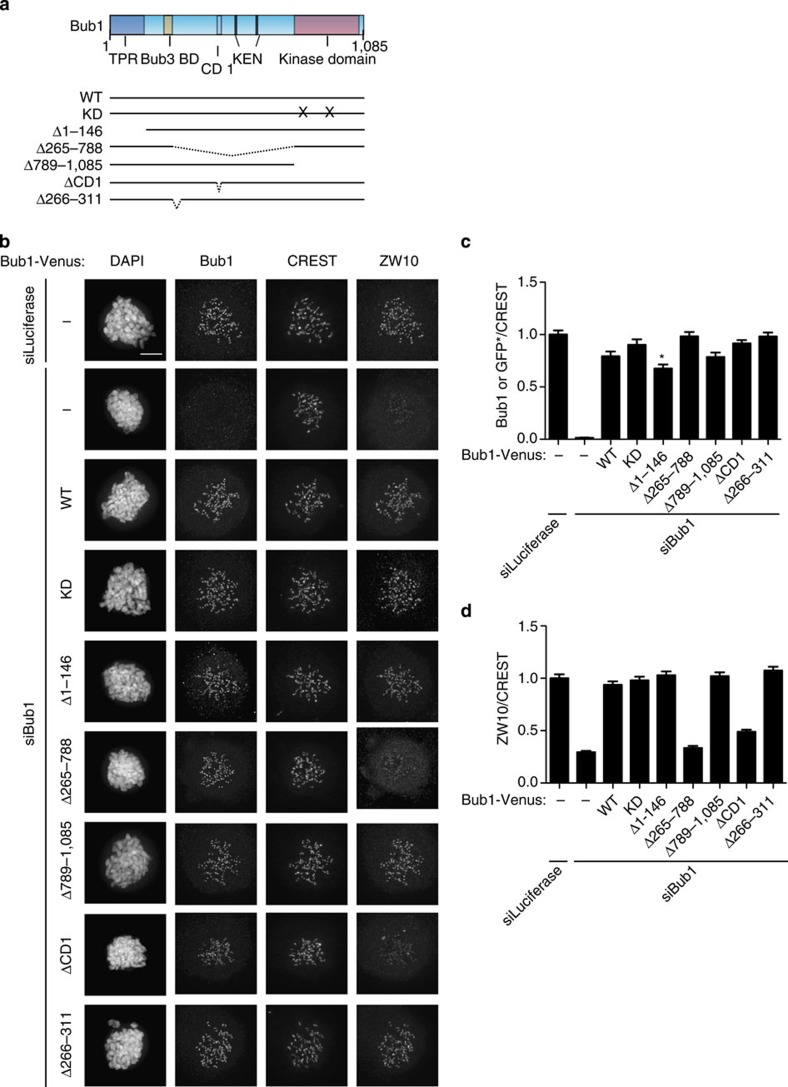
The central region of Bub1 is required for RZZ recruitment. (**a**) Schematic of Bub1 primary structure with motifs indicated and below a schematic of different deletion and mutation constructs used in this study. All Bub1 constructs contain the Bub3 binding site ensuring kinetochore targeting. (**b**) HeLa cells were depleted of endogenous Bub1 using RNAi and the indicated Bub1 constructs were co-transfected for 48 h and cells were arrested with nocodazole for 2 h. Cells were fixed and stained for Bub1, CREST and ZW10. The expression of the exogenous Bub1 construct could be detected by the Bub1 antibody that recognize an epitope in the N terminus of Bub1 except for Bub1 Δ1–146. *To detect Bub1 Δ1–146 expression cells were stained with a GFP antibody. To determine if Bub1 Δ1–146 could recruit ZW10 to kinetochores it was compared with cells expressing wild-type Bub1-Venus at a GFP signal level at which wild-type Bub1 could fully rescue ZW10 localization. The Venus levels were normalized to wild-type Bub1-Venus levels (set to 1) and incorporated into **c**. (**c**,**d**) The Bub1 (**c**) or ZW10 (**d**) intensity at kinetochores was measured and normalized to CREST. At least 160 single kinetochores were analysed from eight different cells and the mean with standard error of mean is indicated. Scale bar, 5 μm.

Bub1 was depleted efficiently to <10% of control levels at kinetochores (see Fig. 3c) in >90% of the cells using our synchronization and depletion protocol (Fig. 1a). Upon Bub1 depletion, there was an ∼65% reduction in ZW10 kinetochore levels while efficient BubR1 depletion resulted in a 30% increase in ZW10 levels (Fig. 2e,f, Supplementary Fig. 3a,b for BubR1 depletion). As BubR1 depletion results in an increase of Bub1 kinetochore levels, these results suggest that Bub1 is clearly needed for ZW10 localization (Supplementary Fig. 3c). We also stained for another RZZ component, Zwilch, and observed a similar effect of Bub1 RNAi (Supplementary Fig. 4a–c). This argues that Bub1 is likely required for recruitment of the entire RZZ complex although we have not been able to test the localization of Rod.

From these results, it is clear that Bub1 is critical for RZZ recruitment. As BubR1 depletion inactivates the checkpoint but does not affect RZZ localiztion, this would argue that RZZ localization does not *per se* depend on an active checkpoint.

### The central region of Bub1 mediates RZZ localization

To determine the regions of Bub1 required for ZW10 kinetochore localization, we generated a panel of Bub1-Venus deletion constructs that were resistant to Bub1 RNAi and contained the Bub3 binding motif ensuring they were targeted to kinetochores (Fig. 3a). We depleted Bub1 and expressed the different Bub1 constructs using the protocol outlined in Fig. 1a. All constructs contained the amino (N)-terminal region recognized by the Bub1 antibody allowing us to compare the relative expression levels of exogenous and endogenous Bub1 (This is except for the N-terminal Bub1 truncation, Bub1 Δ1–146, which removed the antibody epitope and we instead used a GFP antibody—see Fig. 3 legend). Only cells that expressed exogenous Bub1 close to endogenous levels were analysed (Fig. 3c). Next, the ability of the Bub1 proteins to restore ZW10 kinetochore levels was determined (Fig. 3b–d). First, Bub1 wild-type protein fully rescued ZW10 kinetochore levels showing that our complementation assay worked efficiently (Fig. 3b–d). Second, we excluded the possibility that the kinase activity of Bub1 was required as neither mutation of critical residues required for kinase activity (K821R/D946N, referred to as KD (kinase dead)) nor deletion of the entire kinase domain affected ZW10 localization. This was important as it ruled out an indirect effect of Bub1 depletion on ZW10 localization through an effect on Aurora B centromere localization stimulated by Bub1 phosphorylation of T120 on histone H2A^48^. We deleted residues 266–311 from human Bub1 because it did not align to the yeast Bub1 sequence. We suspected that this region might facilitate RZZ localization in higher eukaryotes, as the RZZ complex is absent in yeast. However, Bub1 Δ266–311 fully rescued ZW10 localization as did a Bub1 construct missing the first 145 amino acids. Instead, when we deleted the entire middle region of Bub1, residues 265–788, ZW10 kinetochore levels were not restored and were similar to the levels of the Bub1 RNAi without rescue (Fig. 3b–d). This middle region contains conserved domain I (CD1), residues 458–476, that has been shown to be required for Mad1/2 localization in humans^49^. Indeed Bub1 missing CD1 could only restore ZW10 and Zwilch kinetochore levels to ∼50% of wild-type Bub1 (Fig. 3b–d and Supplementary Fig. 4a–c).

**Figure 4:**
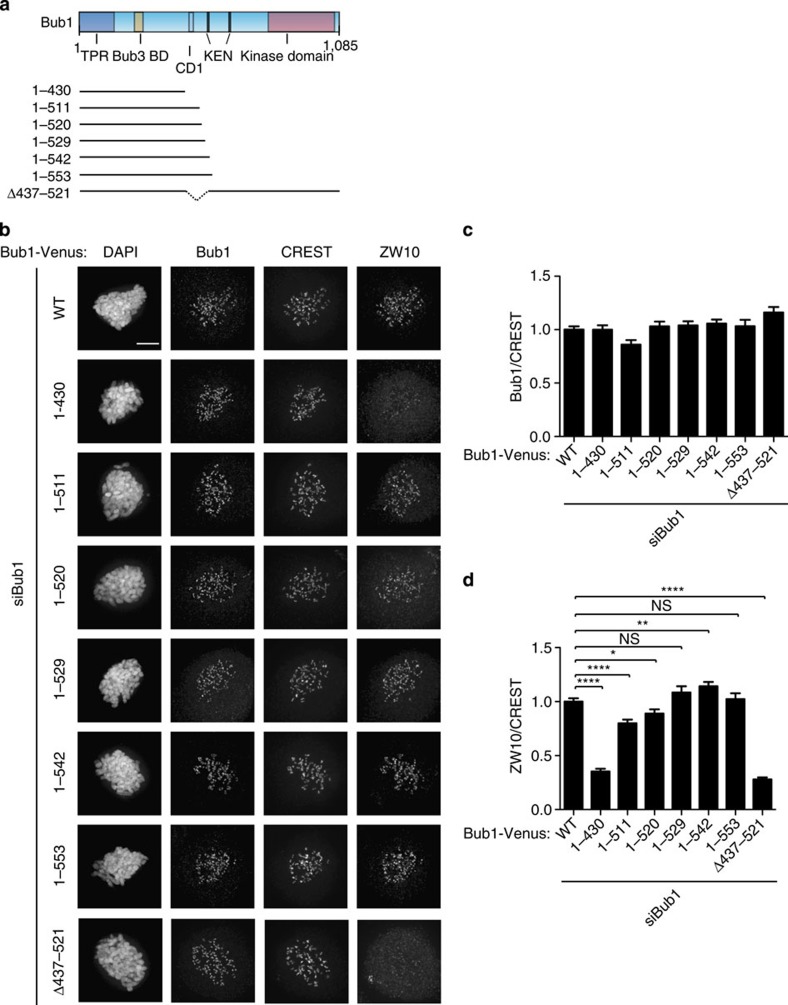
A region encompassing CD1 in Bub1 is required for RZZ localization. (**a**) Schematic of Bub1 primary structure with motifs indicated and below a schematic of the different truncation constructs used in this study. All constructs contain the Bub3 binding site ensuring kinetochore targeting. (**b**) HeLa cells were depleted of endogenous Bub1 using RNAi and the indicated Bub1 constructs were co-transfected for 48 h and cells were arrested with nocodazole for 2 h. Cells were fixed and stained for Bub1, CREST and ZW10. (**c**,**d**) The Bub1 (**c**) or ZW10 (**d**) kinetochores levels was measured and normalized to CREST signals. At least 160 single kinetochores were analysed from eight different cells and the mean with standard error of mean is indicated. A *t*-test was used to compare the values in **d**. (NS, not significant (*P*>0.05), *P≤0.05, **P≤0.01, ****P≤0.0001). Scale bar, 5 μm.

To further delineate which part of the middle region of Bub1 was required for ZW10 localization, we generated additional truncation constructs from the carboxy (C)-terminal end (Fig. 4a). Bub1 1–553 and Bub1 1–529 fully restored ZW10 kinetochore localization similar to wild-type Bub1, while Bub1 1–542 slightly increased ZW10 levels (Fig. 4b–d). Bub1 1–511 and Bub1 1–520 were partially impaired in this and resulted in ∼10–20% reduction of ZW10 levels at kinetochores compared with wild-type Bub1 (Fig. 4b–d). Bub1 1–430 instead did not restore Bub1-dependent ZW10 kinetochore localization. The combined analysis of the truncation constructs thus suggests that residues 430–530 of Bub1 are required for efficient ZW10 kinetochore localization. Indeed, a Bub1 deletion lacking most of this region, Bub1 Δ437–521, did not rescue Bub1-dependent ZW10 or Zwilch kinetochore localization at all (Fig. 4b–d and Supplementary Fig. 4a–c). It is presently not clear whether there is any direct interaction between Bub1 and RZZ components and we have been unable to detect Bub1 in numerous RZZ purifications in agreement with mass spectrometry analysis of these proteins^50^.

To determine whether the role of Bub1 in recruiting Mad1/2 to kinetochores could be mediated via RZZ, we analysed Mad1 localization. We confirmed that the RZZ complex is required for full Mad1 localization as depletion of ZW10 and Rod reduced Mad1 levels by ∼55% (Supplementary Fig. 5a–e). Bub1 RNAi resulted in an ∼40% reduction of Mad1 at kinetochores in agreement with work from the Yu Lab^31^ (Supplementary Fig. 5a–e). Neither Bub1 ΔCD1 nor Bub1 Δ437–521 efficiently rescued Bub1-dependent Mad1 localization (Supplementary Fig. 5f–h). Depletion of Mad1 to 40% of its normal levels at kinetochores did not affect ZW10 kinetochore levels arguing for a linear dependency of Bub1–RZZ–Mad1/2 in localizing Mad1/2 to kinetochores (Supplementary Fig. 6).

In summary, our truncation analysis of Bub1 has identified a central region that is required for Bub1-dependent RZZ localization and this region of Bub1 is also required for the Bub1-dependent localization of Mad1/2 to kinetochores.

### Bub1 localizes BubR1 through direct binding

As Bub1 is both required for RZZ and BubR1 kinetochore localization, it suggested to us that Bub1 could scaffold a large checkpoint complex. We hypothesized that such a large checkpoint complex could catalyse MCC formation. To test this hypothesis, we set out to understand how Bub1 recruits BubR1.

**Figure 5:**
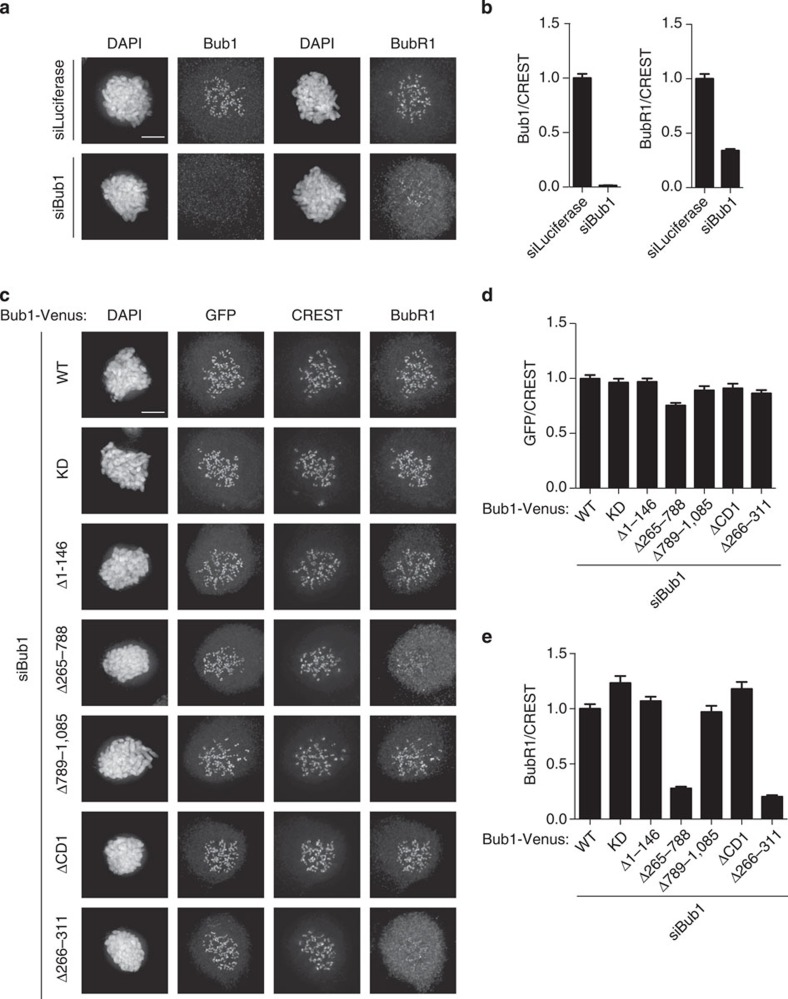
Defining a region in Bub1 required for BubR1 kinetochore localization. (**a**) HeLa cells were treated with a control RNAi oligo (luciferase) or a Bub1 RNAi oligo for 48 h and arrested in mitosis using nocodazole for 2 h. Cells were fixed and stained for CREST and Bub1 or BubR1 separately because both antibodies are mouse monoclonal. (**b**) The kinetochore levels of BubR1 or Bub1 were measured and normalized to CREST. (**c**) HeLa cells were depleted of Bub1 by RNAi and complemented with the indicated RNAi-resistant Bub1 constructs for 48 h and arrested in mitosis using nocodazole for 2 h. Cells were fixed and stained for GFP, CREST and BubR1. First the level of Bub1-Venus, based on the GFP signal that fully restored BubR1 kinetochore levels, was determined. This level of GFP was then used for all constructs. (**d**,**e**) The kinetochore levels of GFP or BubR1 were determined and normalized to CREST. At least 160 single kinetochores were measured from eight different cells and the mean with standard error of mean is indicated. Scale bar, 5 μm.

We used a similar Bub1 RNAi depletion and complementation approach that we used for ZW10 localization. However, due to the fact that both the Bub1 and BubR1 antibodies are mouse monoclonal, we stained for the Venus tag of exogenous Bub1 with a GFP antibody. Bub1 depletion reduced BubR1 kinetochore levels to ∼35% and we then determined the Bub1-Venus levels (based on the Venus signal) that restored BubR1 to normal levels at kinetochores. This level of Venus signal was assumed to be close to endogenous Bub1 levels and used for all the truncation and deletion constructs. Strikingly, two Bub1 deletions were unable to restore any Bub1-dependent BubR1 localization, namely the deletion of the entire middle region, Bub1 Δ265–788, and the deletion of the region from amino acids 266–311 (Fig. 5a–e). To further investigate the residues required in Bub1 for BubR1 kinetochore localization, we made additional shorter deletions in the region from 266 to 311 and analysed BubR1 localization (Supplementary Fig. 7). Except for deletion of amino acids 301–311, which rescued BubR1 kinetochore localization to 75% of Bub1 wild-type levels, all other deletions in this region did not recruit any BubR1 in a Bub1-dependent manner. We thus conclude that the entire region encompassing residues 266–311 of Bub1 is required for efficient BubR1 kinetochore localization and we will refer to this region as the BubR1 localization motif (R1LM).

**Figure 6:**
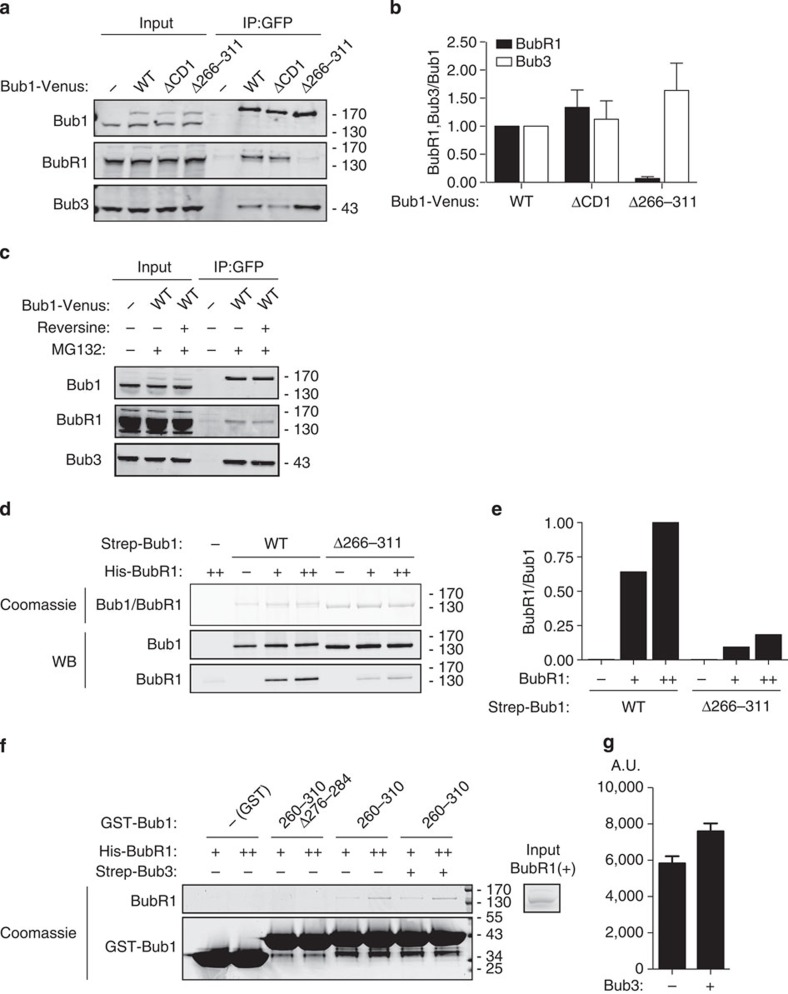
A direct interaction between Bub1 and BubR1. (**a**) HeLa cells were transfected with the indicated Venus-tagged Bub1 constructs and arrested in mitosis with nocodazole and collected by mitotic shake-off. Bub1 complexes were purified using a GFP affinity resin and samples were analysed by western blot for the interaction with BubR1 and Bub3. (**b**) The blots in **a** were quantified using Licor technology. The level of BubR1 and Bub3 normalized to Bub1-Venus is indicated with Bub1 wild-type levels set to 1. The mean with standard deviation is indicated from three independent experiments. (**c**) As in **a**, but cells were treated with the proteasome inhibitor MG132 and the Mps1 inhibitor, reversine, for 30 min before purification of Bub1. Representative of two independent experiments. (**d**) Purified full-length Bub1 or Bub1 Δ266–311 was incubated with purified full-length BubR1 at two different concentrations (+, ++). After incubation, Bub1 proteins were bound to Strep-Tactin beads via their Strep-tag. Following washing, the samples were analysed by Coomasie staining and western blot. Representative of two independent experiments. (**e**) The level of BubR1 in the experiment in **d** was determined using Licor technology and is representative of two independent experiments. The background binding to beads is 6% compared with the maximum binding of BubR1 as observed. (**f**) The indicated GST-tagged protein was immobilized on glutathione beads and incubated with two different concentrations of purified full-length BubR1 (+, ++) and where indicated Bub3 was added. Following incubation, the beads were washed and bound material was eluted. Samples were analysed by SDS–PAGE and Coomasie staining. Representative of three independent experiments. (**g**) The level of BubR1 bound to GST–Bub1 260–310 was measured either in the absence or presence of Bub3. The mean with standard deviation is indicated from three independent experiments. The stimulation of BubR1 binding by Bub3 was determined under low levels (+) of BubR1.

As Bub1 and BubR1 have been shown to interact in human cells^51^, we next determined whether the R1LM of Bub1 was required for this interaction. We expressed exogenous Venus-tagged Bub1, Bub1 Δ266–311 and Bub1 ΔCD1 by transient transfection and purified Bub1 complexes using a GFP affinity resin from nocodazole-arrested cells. The ability of the different Bub1 proteins to interact with BubR1 and Bub3 was investigated by western blot and indeed Bub1 co-purified BubR1 in an R1LM-dependent manner (Fig. 6a,b). We consistently observed that Bub1 Δ266–311 co-purified more Bub3 potentially reflecting some competition between BubR1 and Bub1 for Bub3. To determine whether the Bub1–BubR1 interaction was dependent on an active checkpoint, we inhibited Mps1 using reversine. We purified Bub1-Venus from nocodazole-arrested cells treated with reversine for 30 min and compared this with control-treated cells (Fig. 6c). In these experiments, a proteasome inhibitor, MG132, was added to prevent mitotic exit in reversine-treated cells. Bub1 co-purified almost similar amounts of BubR1 from the reversine- and control-treated cells.

To determine whether there was a direct interaction between Bub1 and BubR1, we expressed and purified full-length Bub1 and Bub1Δ266–311 in HEK293 cells and full-length BubR1 in insect cells. The Bub1 protein was bound to Strep-Tactin beads via their Strep-tag and incubated with two different concentrations of BubR1. Following washing, the bound samples were analysed by Coomasie staining and western blot. The results clearly showed that full-length BubR1 bound to Bub1 and this strongly depended on the R1LM arguing, for a direct interaction (Fig. 6d,e).

To investigate whether the R1LM itself could directly bind BubR1, we purified two GST fusions of Bub1: Bub1 260–310 and Bub1 260–310 (Δ276–284) and analysed the ability of these proteins to bind full-length BubR1. We added two different concentrations of BubR1 to the GST–Bub1 fusions and used GST as a control. Bub1 260–310 could directly bind BubR1 and this binding was lost when we deleted residues 276–284 (Fig. 6f). We measured that ∼5% of the BubR1 in the input bound to GST–Bub1 260–310 in these experiments. We also added Bub3 to see whether this stimulated the interaction, as BubR1 has to be in a complex with Bub3 to localize to kinetochores^18^. The addition of Bub3 stimulated the interaction slightly (Fig. 6g).

Our results show that the ability of Bub1 to recruit BubR1 to kinetochores depends on a direct interaction between BubR1 and the R1LM of Bub1. This interaction might not be restricted to kinetochores as we can detect binding in the presence of reversine and with purified recombinant proteins.

### The CD1 but not the R1LM of Bub1 is essential for the SAC

To investigate whether the regions in Bub1 that were found to be required for RZZ and BubR1 kinetochore localization were critical for SAC signalling, we turned to a complementation assay with our different Bub1 truncations. Bub1 removal has to be almost complete before the SAC is inactivated^52^. Despite our Bub1 depletion being efficient, it did not prevent HeLa cells from maintaining a robust mitotic arrest in response to microtubule poisons. Therefore, we obtained immortalized mouse embryonic fibroblasts (iMEFs) from the Taylor Lab in which Bub1 exon 7 and 8 are flanked by *loxP* sites. Bub1 can be fully inactivated by adding 4-hydroxy-tamoxifen (OHT) to these cells for 24 h (refs 53, 54).

**Figure 7:**
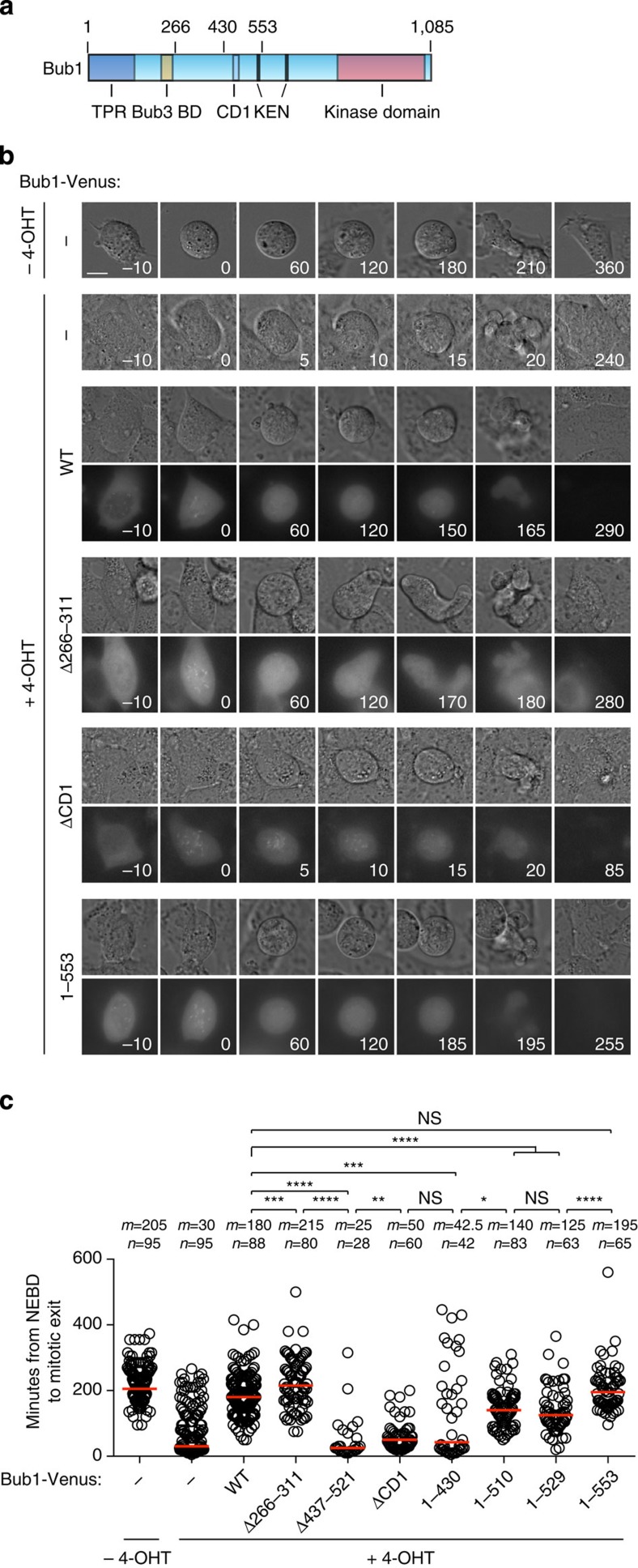
The RZZ interaction domain but not the BubR1 interaction domain is critical for SAC signalling. (**a**) Schematic of Bub1 with domains indicated and the position of specific amino acids marking the boundaries of Bub1 truncations and deletions. (**b**) Immortalized mouse embryonic fibroblasts (iMEFs) where exon 7 and 8 can be loxed out by addition of 4-OHT were analysed by time-lapse microscopy in the presence of 200 nM Taxol. Bub1 was removed by adding 4-OHT for 24 h and where indicated the iMEFs were complemented with human Bub1-Venus constructs by transfection. The differential interference contrast and Venus channels were recorded every 5 min for 18 h. Representative still images are shown for a number of the experimental conditions. The time at nuclear envelope breakdown (NEBD) is set to zero. Scale bar, 15 μm. (**c**) Single cell analysis of the ability of the different Bub1-Venus constructs to complement the removal of Bub1. Each circle represents a single cell analysed and the red line indicates the median (*m*) with the median value also indicated above. The number (*n*) of single cells analysed per condition is indicated above each condition. A Mann–Whitney test was used for statistical comparison of the different samples. (NS, non-significant *P*>0.05, *P≤0.05, **P≤0.01, ***P≤0.001, ****P≤0.0001).

Human and mouse Bub1 are very similar in sequence (Supplementary Fig. 8) and we therefore complemented Bub1 loss in the iMEFs with our human Bub1 constructs by transfection. Mitotic duration was determined by live-cell imaging using differential interference contrast microscopy in the presence of taxol to activate the checkpoint. Due to the presence of taxol, cells bleb when they exit mitosis and we only scored the time of exit in cells that clearly re-attached and excluded the few cells that died. As expected, the iMEFs arrested in a Bub1-dependent manner and expressing human Bub1-Venus efficiently restored the mitotic arrest (Fig. 7 and Supplementary Movie 1). Bub1-Venus localized to kinetochores in prophase and prometaphase as expected and upon mitotic exit Bub1 appeared to be degraded rapidly. Bub1ΔCD1-Venus hardly complemented Bub1 function in this assay, which is in agreement with the reported requirement for this domain in both yeast and humans for checkpoint activity^49^,^55^. These results supported that our assay accurately reported on the functionality of Bub1. We then proceeded to analyse our Bub1 deletions and as expected Bub1 Δ437–521 did not have any checkpoint activity left. Surprisingly, the removal of the R1LM did not impair checkpoint signalling but instead increased the time spent in taxol, arguing that Bub1-dependent BubR1 kinetochore localization is not required for a functional checkpoint.

Analysis of the different truncation constructs revealed that only Bub1 1–553 complemented Bub1 function fully. Bub1 1–430 did not support checkpoint activity in agreement with the results of the Bub1 Δ437–521 deletion (Fig. 7). We note that Bub1 1–553 was better at restoring checkpoint activity compared with Bub1 1–529 and Bub1 1–511 and this might be due to the presence of Cdc20 interacting motifs present in 530–553 of Bub1 (refs 56, 57).

The analysis of the different Bub1 constructs reveals that the central region that was identified as critical for RZZ localization is required for SAC signalling, while Bub1-dependent BubR1 recruitment is dispensable for the SAC.

## Discussion

Understanding how the checkpoint proteins interact with kinetochores is important for determining how microtubule attachment is sensed by the checkpoint and how the signal is specifically generated at kinetochores. Here, we have provided novel insight into how the BubR1 and RZZ complexes are recruited by distinct regions of Bub1, which reveals how Bub1 acts as a scaffold for regulating SAC signalling.

Previous work has implicated Zwint as the kinetochore receptor for RZZ through a direct interaction between ZW10 and Zwint^44^,^45^. Here, we show that in the context of the kinetochore, Zwint is unable to recruit RZZ by itself. This is in agreement with the identification of ZW10 mutants that do not bind Zwint yet still localize to kinetochores^46^. Our data suggest that if KNL1 is not bound to Zwint then it becomes unstable and targeted for degradation. Recent observations suggest that the yeast KNL1 homologue Spc7 is under tight protein quality control and structurally perturbed Spc7 mutants get targeted for degradation^58^. The Zwint binding domain on KNL1 overlaps with an coiled-coil region that precedes an RWD domain^47^,^59^. Proteins with coiled-coil and RWD domains often forms homo- or heterodimers but KNL1 is monomeric^59^. Zwint could, therefore, be the binding partner for KNL1 in lieu of homodimerization. Removal of Zwint might then affect the structural integrity of KNL1 and target it for degradation by a protein quality control pathway.

We show that a central region spanning residues 437–521 of Bub1 is needed for efficient RZZ kinetochore localization and for checkpoint signalling. This is consistent with the nanometre-scale mapping from the Salmon Lab that placed RZZ subunits close to the N terminus of KNL1 and to Bub1 in metaphase-arrested cells where dynein stripping is prevented^7^. At the moment, we do not know whether Bub1 can directly bind the RZZ complex, as we have been unable to detect Bub1 in RZZ purifications. Either the interaction is very labile and lost during purification, or additional Bub1-dependent bridging factors are involved. As some RZZ remains at kinetochores after efficient depletion of Bub1, additional contacts between the kinetochore and the RZZ complex possibly exist that together with Bub1 could generate a combined binding site for RZZ. Our data also do not rule out the possibility that once the RZZ complex is localized, a Zwint–ZW10 interaction could help stabilize the complex on kinetochores as reported^46^.

**Figure 8:**
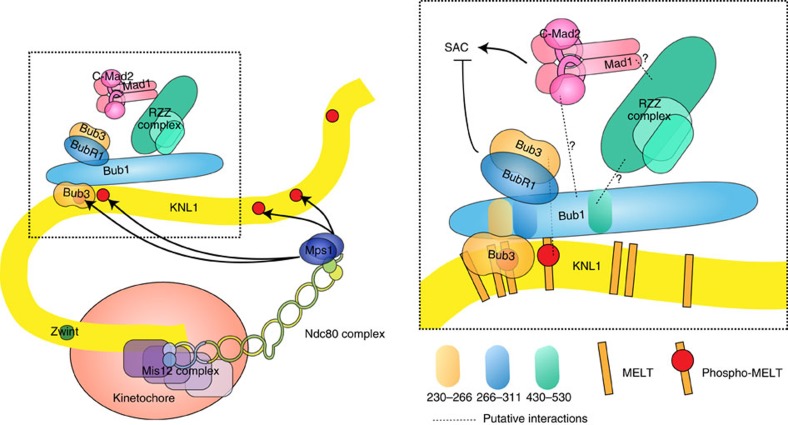
Model for the recruitment of RZZ, Mad1/2 and BubR1 to kinetochores. Schematic of the KMN network with Mps1 binding to Ndc80 to phosphorylate MELT repeats in KNL1 to create a docking site for Bub1–Bub3. Bub1–Bub3 recruits RZZ through its central domain and this stimulates Mad1/2 recruitment. BubR1–Bub3 is recruited through its interaction with the R1LM of Bub1 spanning residues 266–311. Dotted lines indicate putative interactions that might not be direct.

As the RZZ complex is required for efficient Mad1/2 localization our finding that Bub1 is required for RZZ localization could explain why Bub1 contributes to Mad1/2 localization^16^,^17^,^31^. Indeed, deletion of residues 437–521 from Bub1 prevents both Bub1-dependent RZZ and Bub1-dependent Mad1/2 localization. This would argue that the role of Bub1 in Mad1/2 kinetochore localization is mediated through RZZ. As Mad1 depletion does not affect ZW10 levels, we favour a linear Bub1–RZZ–Mad1/2 pathway of dependencies. It is, however, clear that Bub1 must have an important role in the checkpoint beyond Mad1/2 recruitment because in cells where Mad1 is artificially recruited to kinetochores Bub1 is still required for SAC signalling^55^,^60^. A direct Bub1–Mad1/2 (albeit weak) interaction could occur at kinetochores that is critical for SAC signalling but dispensable for Mad1/2 localization (Fig. 8, ref. 61).

It is interesting that the central region what was identified in Bub1 as required for RZZ localization, parallels the observations from the Biggins Lab that identified a central region of yeast Bub1 binding directly to Mad1/2 to localize the complex to kinetochores^32^. This might suggest that the yeast Bub1–Mad1/2 interaction was replaced with a Bub1–RZZ–Mad1/2 pathway in more complex eukaryotes during evolution.

We also uncover how Bub1 recruits BubR1 to kinetochores through a direct interaction between the R1LM of Bub1 and BubR1 in agreement with recent observations from the Musacchio Lab, which was published during revision of this work^62^. Although we show that the interaction between BubR1 and the R1LM can occur independently of Bub3, it is clear that BubR1 needs to be bound to Bub3 to localize to kinetochores *in vivo*^18^. It is likely that at *in vivo* concentrations of the proteins, this role of Bub3 becomes essential. Potentially, when BubR1 binds the R1LM of Bub1, this allows Bub3 (bound to BubR1) to bind to phosphorylated MELT repeats of KNL1 to further enhance BubR1 kinetochore localization (Fig. 8). Alternatively, the role of Bub3 is to present BubR1 for Bub1 binding by affecting the folding of BubR1. As Bub1 is a more stable component of the kinetochore compared with BubR1, we envision that the interaction between Bub1 and BubR1 is regulated^3^. Future work needs to address the role of Bub3 in localizing BubR1 and the regulation of the BubR1–Bub1 interaction.

Interestingly, preventing Bub1-dependent BubR1 kinetochore localization by removing the R1LM of Bub1 did not prevent SAC signalling but rather increased its strength. This suggests that BubR1 bound to Bub1 is not recruited to kinetochores for SAC signalling but rather for silencing. This is in line with a number of recent observations showing that B56–PP2A phosphatase complexes bound to BubR1 promotes SAC silencing^27^,^28^.

Is there then any role of kinetochore localized BubR1 in generating the SAC signal? As some BubR1 remains after efficient Bub1 depletion and as two kinetically distinct pools of BubR1 have been observed^3^, it is possible that these pools of BubR1 have distinct functions in the checkpoint. Alternatively, the rate-limiting step in generating the MCC is the binding of Mad2 to Cdc20 that is catalysed at kinetochores and then BubR1 subsequently binds in the cytoplasm^21^,^22^,^23^. Ongoing efforts in the lab are focused on investigating these models.

## Methods

### Cell culture

HeLa cells (ATCC) were cultivated in DMEM medium (Invitrogen) supplemented with 10% fetal bovine serum and antibiotics. Cells were synchronized with 2 mM thymidine for 24 h before co-transfection with short interfering RNA oligos (50 nM as final concentration) and rescue constructs by Lipofectamine 2000 (Life Technologies). RNAi oligos targeting Bub1 (5′-GAGUGAUCACGAUUUCUAA-3′), KNL1 (5′-UUUCGUGGAUCCUUAAUCAGAUCUU-3′), Zwint (5′-GGAGGACACUGCUAAGGGU-3′ ), BubR1 (5′-GAUGGUGAAUUGUGGAAUA-3′ ), Luciferase (5′-CGUACGCGGAAUACUUCGA-3′ ), Mad1 (5′-CGAUUGUGAAGAACAUGAA-3′), Rod (5′-CCACCAUAGUGUUCCGAAU-3′) and ZW10 (5′-UGAUCAAUGUGCUGUUCAA-3′) were used for RNAi depletions. Twelve hours after the transfection, the cells were arrested again by thymidine for another 24 h. The cells were released from thymidine and treated with nocodazole (200 ng ml^−1^) for 2 h when the majority of cells entered mitosis and fixed.

### Cloning

Full-length Bub1 and all the truncated constructs were amplified by PCR with KpnI and NotI sites in the forward and reverse primer, respectively. The PCR products were cloned into the KpnI and NotI sites on pcDNA5/FRT/TO c-Venus vector. Bub1 RNAi resistance was achieved by mutating 5′-GGAACGAAGAGTGATCACGATTTCTAAATCAGAATATTCTG-3′ (621–661) into 5′-GGAACGAAGAGTCATCACCATCTCCAAATCAGAATATTCTG-3′ using whole plasmid PCR with the following primer: 5′-GGAACGAAGAGTCATCACCATCTCCAAATCAGAATATTCTGTGC-3′. The following primers were used to create Bub1 wild type and deletions: Bub1 forward primer (5′-CGATGGTACCACCATGGACACCCCGG-3′), Bub1 reverse primer (5′-CATAGCGGCCGCGCTTTTCGTGAACGC-3′), Bub1 Δ266–311 (5′-CGGAGAAAGCATCCCGCTTCCCAG-3′; whole plasmid PCR), Bub1 Δ266–788 (5′-CGGAGAAAGCATGTCCATCACCTTCTTGG-3′; whole plasmid PCR), Bub1 Δ1–146 forward primer (5′-CGATGGTACCACCATGCTCACTGAAACCC-3′), Bub1 Δ789–1085 reverse (5′-CATAGCGGCCGCGCATAGACCAGCTTAG-3′), Bub1 Δ437–521 (5′-GCCAACACAAGTTCTTCTTTGTCATCTGC-3′; whole plasmid PCR). Bub1 KD and Bub1 CD1 mutants were amplified from plasmids provided by Patrick Meraldi and described in ref. 49. KNL1 Zwint binding domain mutation was achieved by mutating 5′-CAGAATGAGAGGGAGAAACTTCAAATAAAGATAGATGAGATGGATAAAATACTTAAGAAGATCGATAACTGC-3′ (6004–6075) into 5′-CAGAATGAGAGGGAGGCAGCTCAAATAAAGAT AGATGAGATGGATAAAATAGCTGCGAAGATCGATAACTGC-3′. Zwint RNAi-resistant DNA was synthesized by GeneArt (Life Technologies) and cloned into pcDNA5/FRT/TO c-Venus vector by KpnI and NotI sites. Bub1 DNA encoding fragment 260–310 was amplified by PCR using (5′-ACGTGGATCCCCAATCAACGGAGAAAGC-3′) and (5′-ACGTGCGGCCGCTTAAACCTCGGACCTTTC-3′) and cloned into pGEX5X-1 using BamHI and NotI sites. Bub1 and Bub1Δ266–311 was cloned into His-OneStrep N-term vector pCPR00053 (LIC-pCEP4) using ligation-independent cloning using 5′-TACTTCCAATCCATGGACACCCCGGAAAATGTCCTTCAG-3′ as forward primer and 5′-TATCCACCTTTACTGTTATTTTCGTGAACGCTTACATTCTAAGAGCAGTAC-3′ as reverse primer to amplify Bub1. BubR1 was cloned into pFASTBAC B using BamHI and StuI. All the constructs have been confirmed by full sequencing. Details of the cloning will be provided upon request.

### Immunofluorescence and quantification

Cells growing on coverslips were washed once with PBS and fixed with 4% paraformaldehyde in PHEM buffer (60 mM PIPES, 25 mM HEPES, pH 6.9, 10 mM EGTA, 4 mM MgSO_4_) for 20 min. Fixed cells were further extracted with 0.5% Triton X-100 in PHEM buffer for 10 min. The antibodies used for cell staining include Bub1 (Abcam, ab54893, 1:400), BubR1 (made in house, 1:400), CREST (Antibodies Incorporated, 15–234, 1:400), GFP (Abcam, ab290, 1:400 or Roche, 11814460001, 1:50), KNL1 (made in house, 1:200), Mad1 (Santa Cruz, sc-65494, 1:200), Mad2 (made in house, 1:400), Zwint (Bethyl, A300-781A, 1:400), ZW10 (Abcam, ab21582, 1:200) and Zwilch (kindly provided by Andrea Musacchio). All the fluorescent secondary antibodies are Alexa Fluor Dyes (Invitrogen, 1:1,000). Z-stacks 200 nm apart were recorded on a Deltavision Elite microscope (GE Healthcare) using a × 100 oil objective followed by deconvolution before quantification. Protein intensity on kinetochores was quantified by drawing a circle closely along the rod-like CREST staining covering the interested outer kinetochore protein staining on both ends. The intensity values from the peak three continuous stacks were subtracted of the background from neighbouring areas and averaged. The combined intensity was normalized against the combined CREST fluorescent intensity.

### MEF experiments

Live-cell analysis was performed on a Deltavision Elite system using a × 40 oil objective (GE Healthcare). Cells were seeded in eight-well Ibidi dishes (Ibidi) in advance and before filming, the media was changed to Leibovitz's L-15 (Life Technologies). Appropriate channels were recorded for 18 h and data analysed using Softworx (GE Healthcare). MEFs were transfected with Bub1-Venus constructs by electroporation using the Neon Transfection System (Invitrogen) according to manufacturer's instruction. A total 1 μM 4-OHT and 200 nM taxol were added 24 and 2 h before filming, respectively. Statistical analysis was done using Prism software.

### Immunopurification of complexes

A total 1 × 10^7^ HeLa FRT cells were seeded on day 1, 96 h before collection, pre-synchronised twice with 2.5 mM thymidine overnight for 16 h on day 2 and day 3, then released on day 4 into media containing 330 nM nocodazole for 16 h overnight before collection. The cells were transfected with Bub1 constructs between thymidine blocks on day 3 using lipofectamine 2000 (Life Technologies). The mitotic population was collected by shake-off, washed once in ice-cold PBS and cell pellets lysed on ice for 45 min in 500 μl lysis buffer (25 mM Tris-HCl pH 7.4, 150 mM NaCl, 1 mM EDTA, 10% glycerol, 0.1% NP-40, supplemented with phosphatase inhibitor and protease inhibitor cocktails (both Roche)) followed by passage 10 times through a needle. Lysate was cleared by centrifugation at 21,000*g* and 1 mg cleared lysate added to 40 μl GFP-Trap_A beads (Chromotek) and incubated with for 2 h at 4 degrees with rotation.

Affinity purified complexes were washed three times with 500 μl complete lysis buffer and eluted with 50 μl 3 × LDS lysis buffer. Samples were analysed by western blot using the following antibodies: BubR1 (home-made, 1:1,000), Bub1 (AbCam ab54893, 1:500) and Bub3 (BD Transduction, 611731, 1:500). Uncropped versions of blots appear in Supplementary Fig 9.

### Expression and purification of proteins

Expression of GST-tagged protein was done by transforming BL21(DE3) cells with the pGEX plasmids and then inducing the expression of the protein at 37 degrees for 3 h with 0.5 mM IPTG. Cells were lysed using a high-pressure homogenizer (Avestin) and centrifuged at 20,000*g* for 30 min. The lysate was incubated with glutathion beads (GE Healthcare) for 60 min and washed with 50 column volumes of 250 mM NaCl, 50 mM Tris pH 8.0, 5% glycerol, 5 mM beta-mercaptoethanol. GST protein bound to beads was stored at 4 degrees.

Strep-His-Bub1 and Bub1 Δ266–311 were expressed in HEK293 6E cell lines by transfection with 100 μg ml^−1^ polyethylenimine ‘MAX'(PEI) (polysciences). After 3 days, the Bub1 protein was affinity purified using a Strep-tag/Strep-Tactin purification system (IBA) according to manufacturer's description. The protein was dialysed overnight into 150 mM NaCl, 25 mM Tris-HCl pH 8.0, 5% glycerol, 5 mM beta-mercaptoethanol and stored at 4 degrees.

His-BubR1 was expressed in insect cells using the pFastBac system (Invitrogen). High Five cells were infected with virus in plates and collected after 48 h. The cells were lysed using a nitrogen cavitation bomb and BubR1 purified using Ni-affinity column and eluted with imidazole-containing buffer.

### Binding of purified proteins

Full-length BubR1 (1 or 2 μg) was incubated with 20 μl of glutathione beads pre-bound with ∼25 μg GST fusion proteins in 200 μl PBST. The samples were mixed for 30 min at 24 degrees and afterwards washed with 500 μl PBST three times. A total 50 μl 2XLDS loading buffer was added and samples were boiled and analysed by SDS–PAGE.

Full-length BubR1 (2 or 4 μg) and Bub1 or Bub1Δ266–311 were incubated 10 min on ice in 200 μl PBST before adding to 20 μl of Strep-Tactin beads (IBA) and then incubated an additional 30 min with shaking. The beads were washed with 500 μl PBST three times and proteins eluted with 50 μl 2XLDS loading buffer and analysed by SDS–PAGE.

## Additional information

**How to cite this article:** Zhang, G. *et al*. Distinct domains in Bub1 localize RZZ and BubR1 to kinetochores to regulate the checkpoint. *Nat. Commun.* 6:7162 doi: 10.1038/ncomms8162 (2015).

## Supplementary Material

Supplementary Information Supplementary Figures 1-9

Supplementary Movie 1 Mouse embryonic fibroblasts where endogenous Bub1 has been removed and cells complemented with human Bub1-Venus. Cells were monitored by time-lapse microscopy with 5 minute intervals
