# Supplementary material for: Distinct domains in Bub1 localize RZZ and BubR1 to kinetochores to regulate the checkpoint
Source: Nat Commun. 2015 Jun 2;6:7162. doi: 10.1038/ncomms8162 (PMC4458899; doi:10.1038/ncomms8162)
Supplement: Supplementary Information — Supplementary Figures 1-9 [file ncomms8162-s1.pdf]

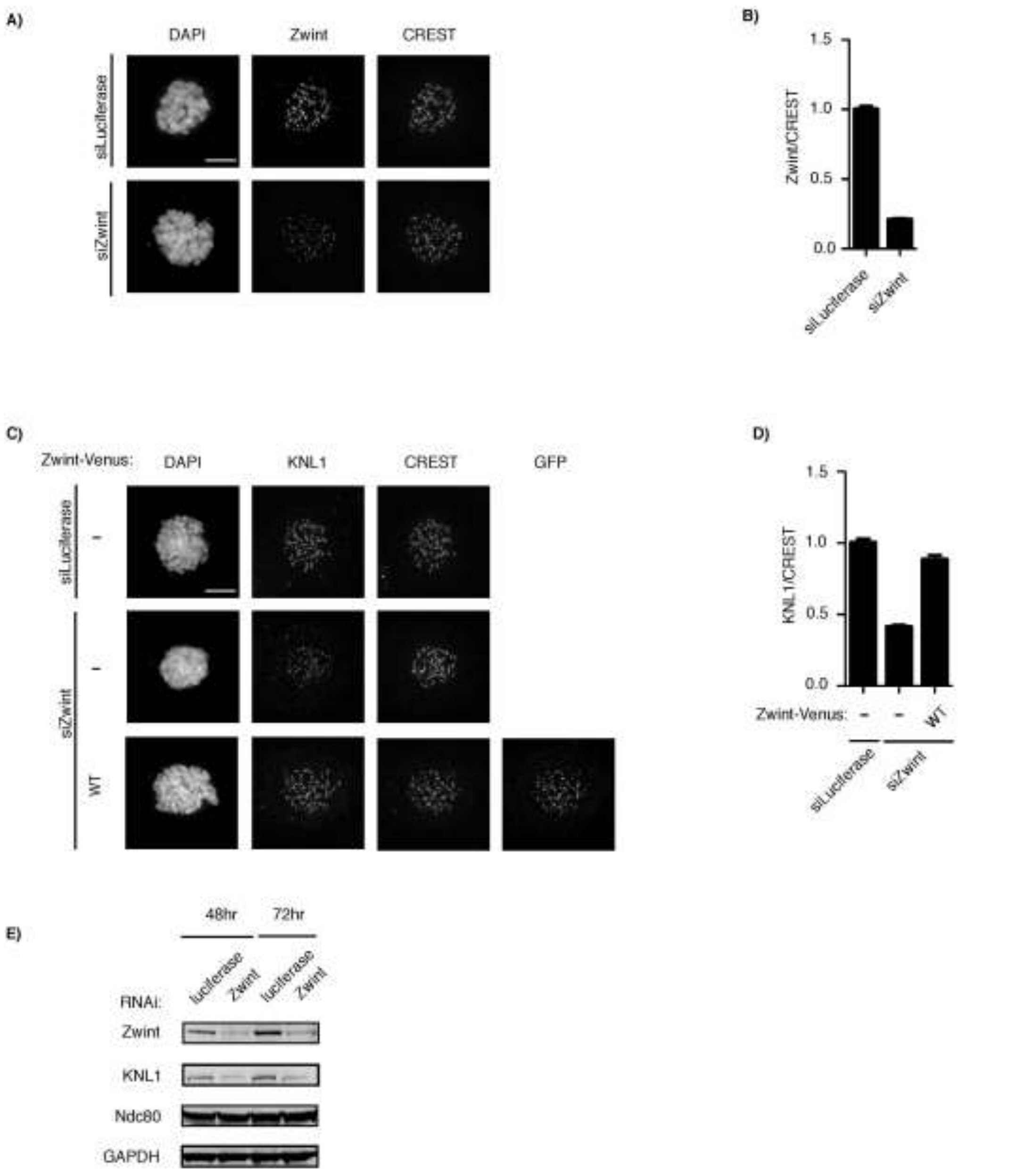

Zhang et al., Supplementary Figure 1

Supplementary Figure 1. Zwint is required for KNL1 localization at kinetochores

A) HeLa cells were treated with luciferase or Zwint RNAi for 48 hours and arrested for 2 hours in mitosis using nocodazole. Cells were fixed and stained for CREST and Zwint. Scale bar, 5 μm. B) The kinetochore levels of Zwint were measured and normalized to

CREST. C) HeLa cells were treated with luciferase or Zwint RNAi for 48 hours and arrested for 2 hours in mitosis using nocodazole. Cells were fixed and stained for CREST, KNL1 and GFP. Where indicated an siRNA resistant Zwint-Venus construct was co-transfected with the Zwint RNAi. Note that since both the Zwint and KNL1 antibodies are rabbit polyclonal antibodies we could not co-stain for the two proteins. Scale bar, 5 μm. D) The kinetochore levels of KNL1 were measured and normalized to CREST. E) HeLa cells treated with luciferase or Zwint RNAi oligoes for 48 hours or 72 hours and arrested in mitosis with nocodazole were collected. Total cell extract was prepared and analyzed by western blot for the levels of Zwint, KNL1, Ndc80 and GAPDH. At least 160 single kinetochores from 8 different cells were measured and the mean with standard error of mean is indicated in A-D).

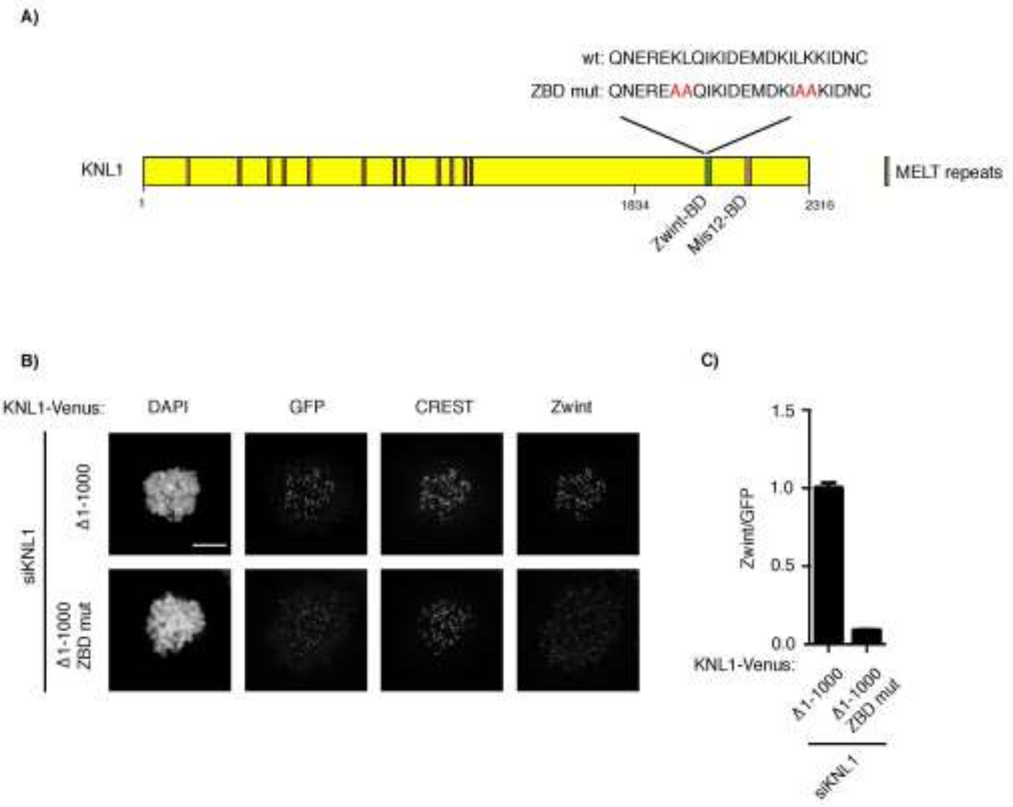

Zhang et al., Supplementary Figure 2

Supplementary Figure 2. Mutation of the Zwint binding domain on KNL1 delocalizes Zwint from kinetochores.

A) Schematic of KNL1 with indication of the mutations in the Zwint binding domain (ZBD) introduced to generate KNL1 ZBD mut. B) HeLa cells were depleted of KNL1 by RNAi for 48 hours and the indicated KNL1-Venus constructs were co-transfected. Cells were arrested in mitosis with nocodazole for 2 hours before fixation. Fixed cells were stained for GFP, CREST and Zwint. Scale bar, 5 μm. C) The kinetochore levels of Zwint were measured and normalized to the GFP signal. At least 160 single kinetochores from 8 different cells were measured and the mean with standard error of mean is indicated.

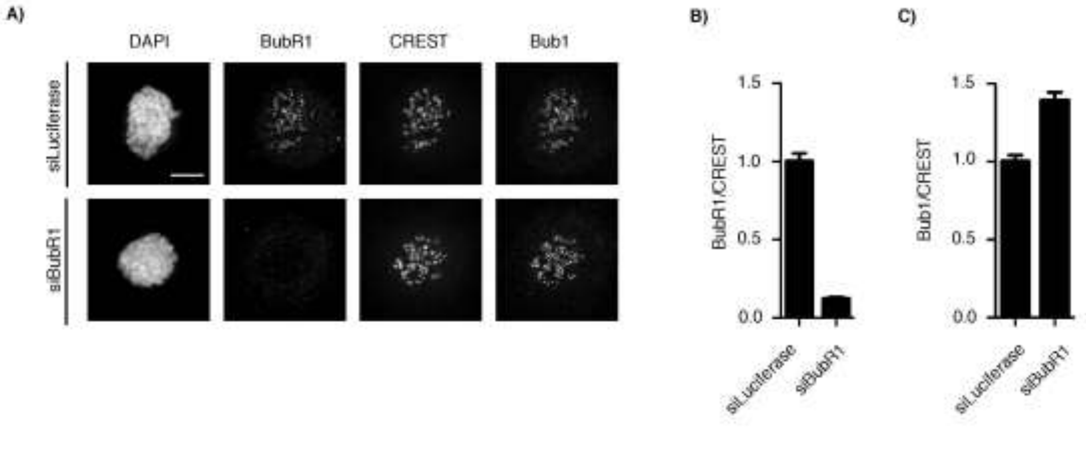

Zhang et al., Supplementary Figure 3

Supplementary Figure 3. Bub1 kinetochore localization increases in BubR1 depleted cells.

A) HeLa cells were treated with luciferase or BubR1 RNAi for 48 hours and arrested in mitosis with nocodazole for 2 hours in the presence of MG132. Cells were fixed and stained for BubR1, Bub1 and CREST. Note: this BubR1 rabbit antibody was also used in Supplementary figure 5 gave very high background with our Alexa Fluor 647 secondary antibody and was not used in the Bub1 domain mapping experiments. Scale bar, 5 μm. B-

C) The kinetochore levels of BubR1 (B) or Bub1 (C) were measured and normalized to CREST. At least 160 single kinetochores from 8 different cells were analyzed and the mean with standard error of mean is indicated.

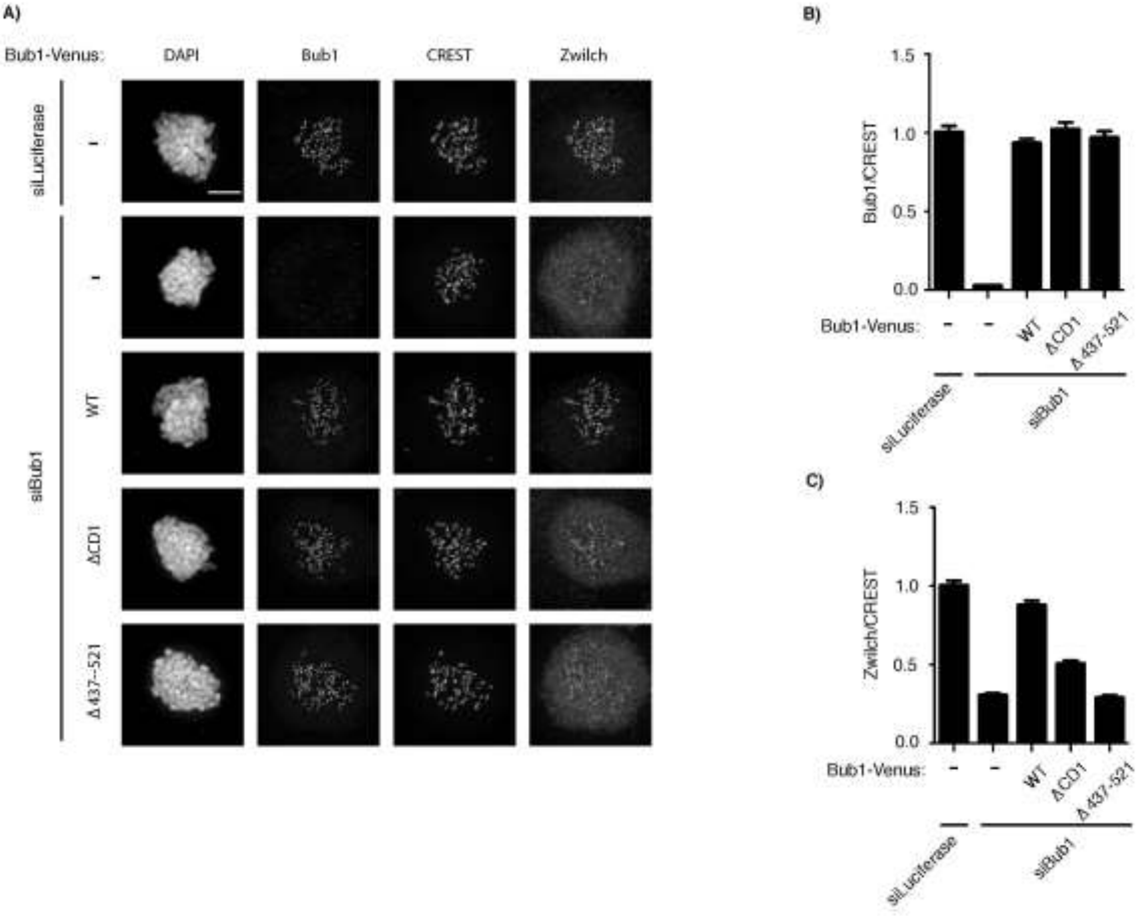

Zhang et al., Supplementary Figure 4

Supplementary Figure 4. The central region in Bub1 contributes to Zwilch localization.

A) HeLa cells were treated with luciferase or Bub1 RNAi and complemented with the indicated RNAi resistant Bub1-Venus constructs by co-transfection for 48 hours. Cells were then arrested in mitosis with nocodazole for 2 hours. Cells were fixed and stained for Bub1, CREST and Zwilch. Scale bar, 5 µm. B-C) The Bub1 (B) or Zwilch (C) intensity at kinetochores was measured and normalized to the CREST signal. At least 160 single

kinetochores were analyzed from 8 different cells and the mean with standard error of mean is indicated.

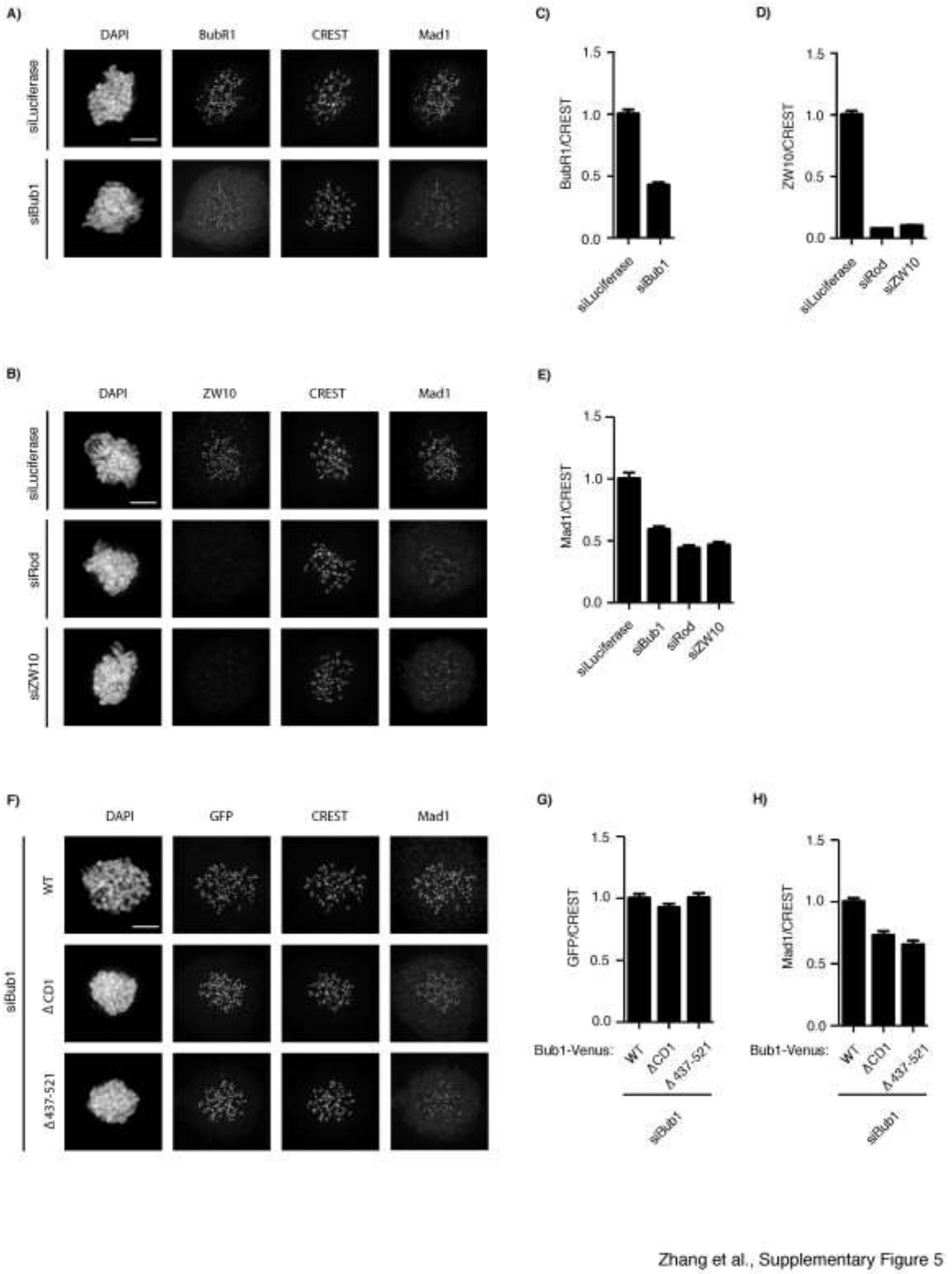

Supplementary Figure 5. Bub1 and RZZ are required for efficient Mad1-Mad2 recruitment to kinetochores.

A-B) HeLa cells were treated with luciferase, Bub1, Rod an ZW10 oligoes as indicated for 48 hours and arrested in mitosis for 2 hours with nocodazole. Cells were fixed and stained for CREST, Mad1, BubR1 and ZW10 as indicated. In A), BubR1 antibody was used as an indicator for efficient Bub1 depletion due to the fact both Bub1 and Mad1 antibodies are mouse monoclonal antibody. In B), ZW10 antibody was used as an indicator for efficient Rod and ZW10 depletion due to the lack of qualified Rod antibody. C) The kinetochore levels of BubR1 were measured and normalized to CREST to examine the depletion of Bub1. D) The kinetochore levels of ZW10 were measured and normalized to CREST to examine the depletion of Rod and ZW10. E) The kinetochore levels of Mad1 were measured and normailzed to CREST in the indicated conditions. F) HeLa cells were treated with Bub1 RNAi and complemented with the indicated Bub1 RNAi resistant Bub1-Venus constructs by co-transfection for 48 hours and arrested in mitosis with nocodazole for 2 hours. Cells were fixed and stained for GFP, CREST and Mad1. G) The kinetochore levels of GFP were measured and normalized to CREST. H) The kinetochore levels of Mad1 were measured and normalized to CREST. At least 160 single kinetochores from 8 different cells were analyzed per condition and the mean with standard error of mean is indicated. Scale bar, 5 μm.

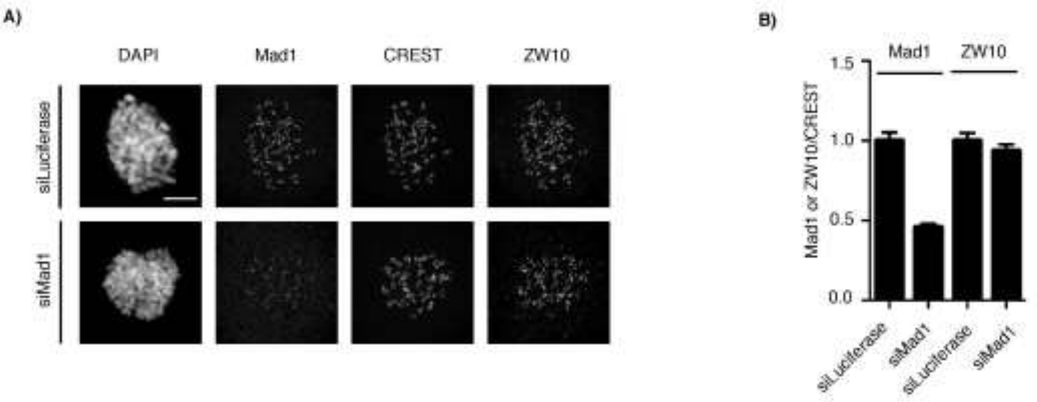

Zhang et al., Supplementary Figure 6

Supplementary Figure 6. Mad1 is not required for ZW10 recruitment to kinetochores. A) HeLa cells were treated with luciferase or Mad1 RNAi for 48 hours and arrested in mitosis with nocodazole for 2 hours. Cells were fixed and stained for Mad1, CREST and ZW10. Scale bar, 5 µm. B) The kinetochore levels of Mad1 and ZW10 were measured and normalized to CREST. At least 160 single kinetochores from 8 different cells were measured and the mean with standard error of mean is indicated.

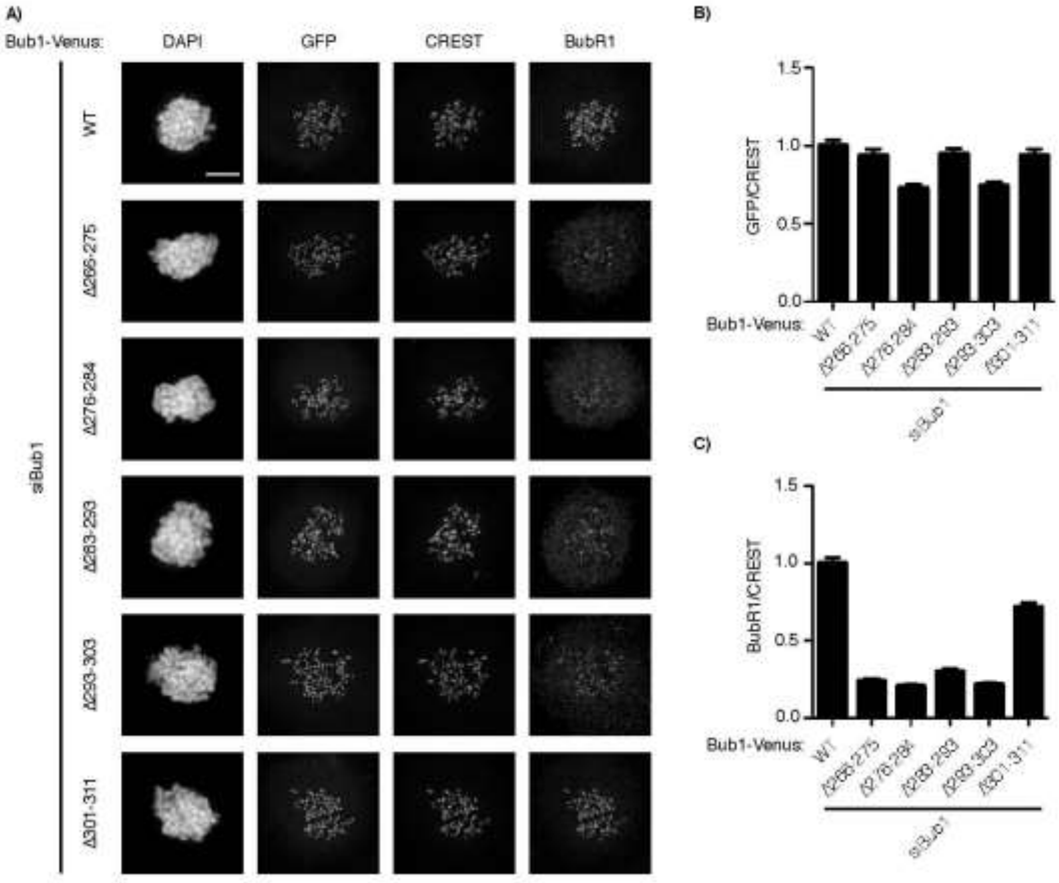

Zhang et al., Supplementary Figure 7

Supplementary Figure 7. Fine mapping of the region in Bub1 required for BubR1 kinetochore localization.

A) HeLa cells were treated with Bub1 RNAi oligoes and complemented with RNAi resistant Bub1-Venus constructs by co-transfection for 48 hours and arrested in mitosis with nocodazole for 2 hours. Cells were fixed and stained for GFP, CREST and BubR1. Scale bar, 5 μm. B) The kinetochore levels of GFP were measured and normalized to CREST. C) The kinetochore levels of BubR1 were measured and normalized to CREST. At least 160 single kinetochores were measured from 8 different cells and the mean with standard error of mean is indicated per condition.

```
1       MDTPENVLQMLEAHMQSYKGNDPLGEWERYIQWVEENFPENKEYLITLLEHLMKEFLDKKKYHNDPRFISYCLKFAEYNS  80
1       MDNLENVFRMFEAHMQSYTGNDPLGEWESFIKWVEENFPDNKEYLMTLLEHLMKEFLHKKNYHNDSRFINYCLKFAEYNS  80

81     DLHQFFEFLYNHGIGTLSSPLYIAWAGHLEAQGELQHASAVLQRGIQNQAEPREFLQQQYRLFQTRLTETHLPAQARTSE  160
81     DRHQFFEFLYNQGIGTKSSYIYMSWAGHLEAQGELQHASAIFQTGIHNEAEPKELLQQQYRLFQARLTGIHLPAQATTSE  160

161    PLHNVQVLNQMITSKSNPGNNMACISKNQGSELSGVISSACDKESNM-ERRVITISKSEYSVHSSLASKVDVEQVVMYCK  239
161    PLHSAQILNQVMMTNSSPEKNSACVPRSQGSECSGVASSTCDEKSNIREQRVIMISKSECSVSSSVAPKPEAQQV-MYCK  239

            Bub3BD                          R1LM
240    EKLIRGESEFSFEELRAQKYNQRRKHEQWVNEDRHYMKRKEANAFEEQLLKQKMDELHKKLHQVVETSHEDLPASQERSE  319
240    EKLIRGDSEFSFEELRAQKYNQRKKHEQWVSEDRNYMKRKEANAFEEQLLKQKMDELHKKLHQVVELSHKDLPASENRPD  319

320    VNPARMGPSVGSQQELRAPCLPVTYQQTPVNMEKNPREAPPVVPPLANAISAALVSPATSQSIAPPVPLKAQTVTDSMFA  399
320    VSLVCVGQNTCSQQELRGPSLSSISHQTSESSGEKPQEEPS-VPLMVNAVNSTLLFPAANLP-ALPVPVSGQSLTDSR--  395

                                                  RZZLM
400    VASKDAGCVNKSTHEFKPQSGAEIKEGCETHKVANTSSFHTTPNTSLGMVQATPSKVQPSPTVHTKEALGFIMNMFQAPT  479
396    -------CVNQSVHEFMPQCGPETKEVCETNKVASINDFHTTPNTSLGMVQGTPCKVQPSPTVHTKEALGFIMDMFQAPT  468

480    LPDISDDKDEWQSLDQNEDAFEAQFQKNVRSSGAWGVNKIISSLSSAFHVFEDGNKENYGLPQPKNKPTGARTFGERSVS  559
469    LPDISDDKDEWPSLDQNEDAFEAQFQKNAVSSGDWGVKKIM-TLSSAFPIFEDGNKENYGLPQPKNKPLGARTFGERSLS  547

560    RLPSKPKEEVPHAEEFLDDSTVWGIRCNKTLAPSPKSPGDFTSAAQLASTPFHKLPVESVHILEDKENVVAKQCTQATLD  639
548    KYSSR-SNEMPHTDEFMDDSTVCGIRCNKTLAPSPKSIGDFTSAAQLSSTPFHKFPADLVQIPEDKENVVATQYTHMALD  626

640    SCEENMVVPSRDGKFSPIQEKSPKQALSSHMYSASLLRLSQPAAGGVLTCEAELGVEACRLTDTDAAIAEDPPDAIAGLQ  719
627    SCKENIVDLSKGRKLGPIQEK----------ISASLPCPSQPATGGLFTQEAVFGLEAFKCTGIDHATVEDLSDANAGLQ  696

720    AEWMQMSSLGTVDAPNFIVGNPWDDKLIFKLLSGLSKPVSSYPNTFEWQCKLPAIKPKTEFQLGSKLVYVHHLLGEGAFA  799
697    VECVQ--TLGNVNAPSFTVENPWDDELILKLLSGLSKPVTSYSNTFEWQSKLPAIKTKTEYQLGSLLVYVNHLLGEGAFA  774

800    QVYEATQGDLNDAKNKQKFVLKVQKPANPWEFYIGTQLMERLKPSMQHMFMKFYSAHLFQNGSVLVGELYSYGTLLNAIN  879
775    QVFEAIHGDVRNAKSEQKCILKVQRPANSWEFYIGMQLMERLKPEVHHMFIKFYSAHLFKNGSILVGELYSYGTLLNVIN  854

880    LYKNTPEKVMPQGLVISFAMRMLYMIEQVHDCEIIHGDIKPDNFILGNGFLEQDDEDDLSAGLALIDLGQSIDMKLFPKG  959
855    LYKNTSEKVMPQALVLTFAIRMLYMVEQVHSCEIIHGDIKPDNFILGHRFLEQADE-DLATGLALIDLGQSIDMKLFPKG  933

960    TIFTAKCETSGFQCVEMLSNKPWNYQIDYFGVAATVYCMLFGTYMKVKNEGGECKPEGLFRRLPHLDMWNEFFHVMLNIP  1039
934    TVFTGKCETSGFQCPEMLSNKPWNYQIDYFGVAATIYCMLFGSYMKVKNEGGVWKPEGLFRRLPHLDMWEEFFHIMLNIP  1013

1040   DCHHLPSLDLLRQKLKKVFQQHYTNKIRALRNRLIVLLLECKRSRK  1085 (Human)
1014   DCHNLPSLDFLRQNMKKLLEQQYSNKIKTLRNRLIVMLSEYKRSRK  1059 (Mouse)
```

Zhang et al, Supplementary Figure 8

Supplementary Figure 8. Alignment of human and mouse Bub1.

Human (top) and mouse (bottom) Bub1 sequences were aligned with red indicating identical residues. The position of the BubR1 localization motif (R1LM) and RZZ localization motif (RZZLM) are indicated as well as the Bub3 binding domain (Bub3BD). The conserved domain 1 (CD1) is highlighted in yellow.

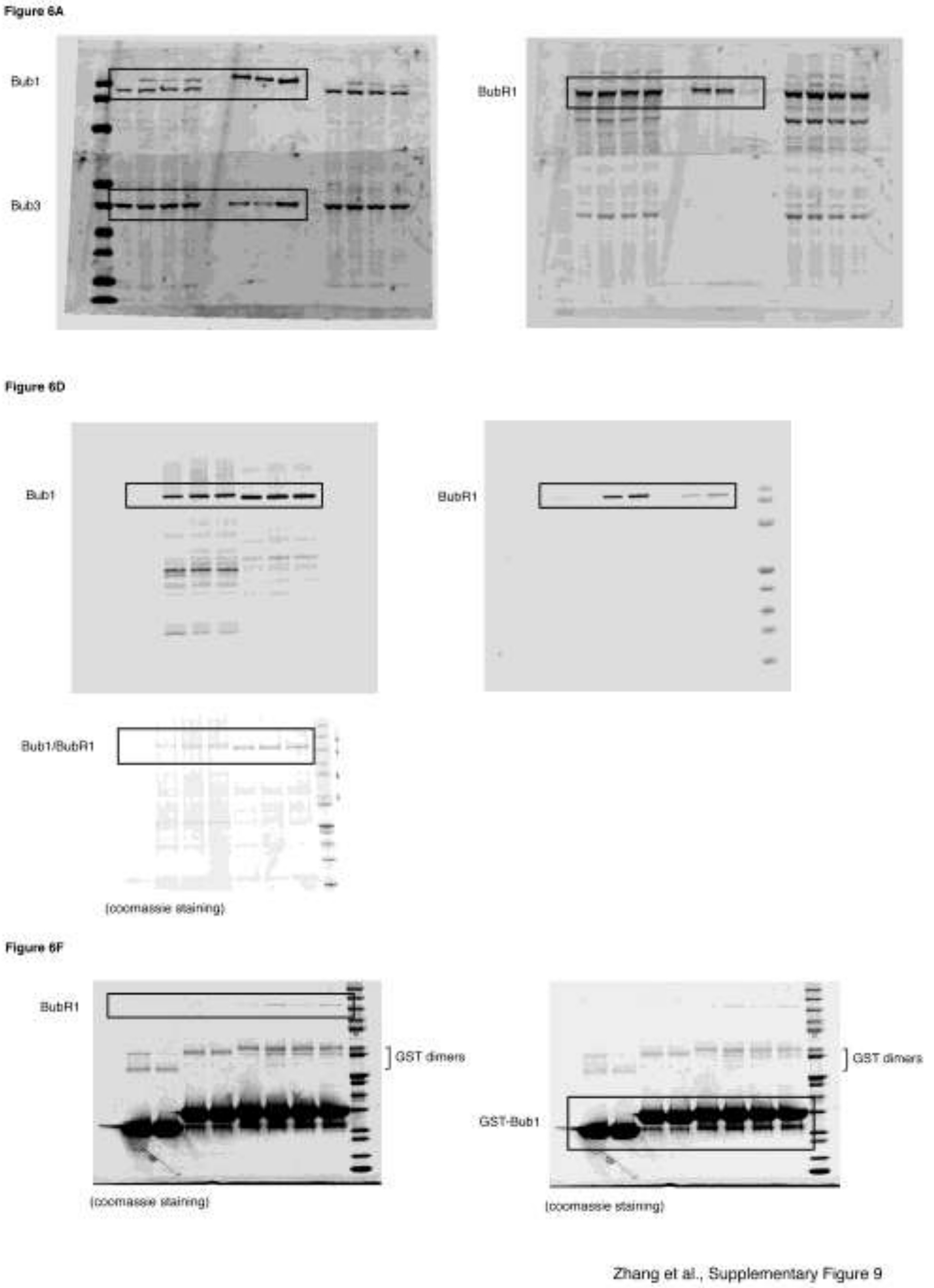

Supplementary Figure 9. Uncropped western blots and Coomasie gels used in Fig. 6.

The cropped regions shown in Fig. 6 are marked with black box on the original blots and gels. The antibody used for staining is indicated and Coomasie gels are also marked.
